# Highly efficient blue organic light-emitting diodes using quantum well-like multiple emissive layer structure

**DOI:** 10.1186/1556-276X-9-191

**Published:** 2014-04-24

**Authors:** Ju-An Yoon, You-Hyun Kim, Nam Ho Kim, Seung Il Yoo, Sang Youn Lee, Fu Rong Zhu, Woo Young Kim

**Affiliations:** 1 Department of Green Energy & Semiconductor Engineering, Hoseo University, Asan 336-795, South Korea; 2 Department of Physics, Hong Kong Baptist University, Hong Kong, China

**Keywords:** Blue organic light-emitting diodes, HOMO-LUMO, QWS

## Abstract

In this study, the properties of blue organic light-emitting diodes (OLEDs), employing quantum well-like structure (QWS) that includes four different blue emissive materials of 4,4′-bis(2,2′-diphenylyinyl)-1,1′-biphenyl (DPVBi), 9,10-di(naphth-2-yl)anthracene (ADN), 2-(*N*,*N*-diphenyl-amino)-6-[4-(*N*,*N*-diphenyl amine)styryl]naphthalene (DPASN), and bis(2-methyl-8-quinolinolate)-4-(phenyl phenolato) aluminum (BAlq), were investigated. Conventional QWS blue OLEDs composed of multiple emissive layers and charge blocking layer with lower highest occupied molecular orbital (HOMO)-lowest unoccupied molecular orbital (LUMO) energy level, and devices with triple emissive layers for more significant hole-electron recombination and a wider region for exciton generation were designed. The properties of triple emissive layered blue OLEDs with the structure of indium tin oxide (ITO) /*N*,*N*′-diphenyl-*N*,*N*′-bis(1-naphthyl-phenyl)-(1,1′-biphenyl)-4,4′-diamine (NPB) (700 Ǻ)/X (100 Ǻ)/BAlq (100 Ǻ)/X (100 Ǻ)/4,7-diphenyl-1,10-phenanthroline (Bphen) (300 Ǻ)/lithium quinolate (Liq) (20 Ǻ)/aluminum (Al) (1,200 Ǻ) (X = DPVBi, ADN, DPASN) were examined. HOMO-LUMO energy levels of DPVBi, ADN, DPASN, and BAlq are 2.8 to 5.9, 2.6 to 5.6, 2.3 to 5.2, and 2.9 to 5.9 eV, respectively. The OLEDs with DPASN/BAlq/DPASN QWS with maximum luminous efficiency of 5.32 cd/A was achieved at 3.5 V.

## Background

Since the report by Tang and VanSlyke on organic light-emitting diodes (OLEDs), [1,2] OLEDs have become a popular research subject due to its several technical advantages such as reduced power consumption, compatibility with flexible substrates, high color rendering index, high contrast, and wide viewing angle. OLEDs have emerged as strong candidates for next-generation flat panel displays and solid-state lighting sources [3-6]. Many progresses have been made in improving the performance of OLEDs, including high power efficiency tandem organic light-emitting diodes based on bulk heterojunction organic bipolar charge generation layer [7]. However, improving the performance of blue OLEDs still remains as an open challenge [8-10]. Various methods have been developed to optimize blue OLED's performance. Such methods include replacing emitters from fluorescent to phosphorescent materials [11], including balancing the carrier ratio in the emissive layer (EML) [12], designing a better surface texture for improving external quantum efficiency [13], and reduced efficiency roll-off in OLEDs at ultrahigh current densities by suppression of triplet-polaron quenching [14].

Among various methods for enhanced efficiency, the QWS has proved to be an effective approach for high device performance [15,16], by confining charge carriers and exciton within the multi-emitting layer. Thus, the charge carrier recombination efficiency and exciton formation probability can be beneficially enhanced [17]. The organic molecules were insufficiently restricted by Van der Waals force among molecules in the organic quantum well. The main features of QWS were high electroluminescence (EL) efficiency [18], tunable EL zone [19], and great carrier balance [20-23].

In this study, the performance of blue OLEDs with multiple emissive layers 4,4′-bis(2,2′-diphenylyinyl)-1,1′-biphenyl (DPVBi), 9,10-di(naphth-2-yl)anthracene (ADN), 2-(*N*,*N*-diphenyl-amino)-6-[4-(*N*,*N*-diphenyl amine)styryl]naphthalene (DPASN), and bis(2-methyl-8-quinolinolate)-4-(phenyl phenolato) aluminum (BAlq) was investigated. These emissive materials have different highest occupied molecular orbital (HOMO)-lowest unoccupied molecular orbital (LUMO) energy levels. Emissive layers with different orders in the QWS-type OLEDs were investigated and optimized to achieve the best device performances. Luminous efficiency and *I*-*V*-*L* characteristics were observed considering the effects of QWS and the variation of recombination region in EML.

### Experiment

Indium tin oxide (ITO)-coated glass was cleaned in ultrasonic bath by regular sequences: in acetone, methanol, diluted water, and isopropyl alcohol. Hereafter, pre-cleaned ITO was treated by O_2_ plasma under condition of 2 × 10^-2^ Torr and 125 W for 2 min. Blue OLEDs were fabricated using the high vacuum (1.0 × 10^-6^ Torr) thermal evaporation and *N*,*N*′-diphenyl-*N*,*N*′-bis(1-naphthyl-phenyl)-(1,1′-biphenyl)-4,4′-diamine (NPB), BAlq, DPVBi, ADN, DPASN, 4,7-diphenyl-1,10-phenanthroline (Bphen), lithium quinolate (Liq), and aluminum (Al) were deposited at different evaporation rates of 1.0, 0.5, 0.5, 0.5, 0.5, 1.0, 0.1, 5.0 Ǻ/s.

**Figure 1:**
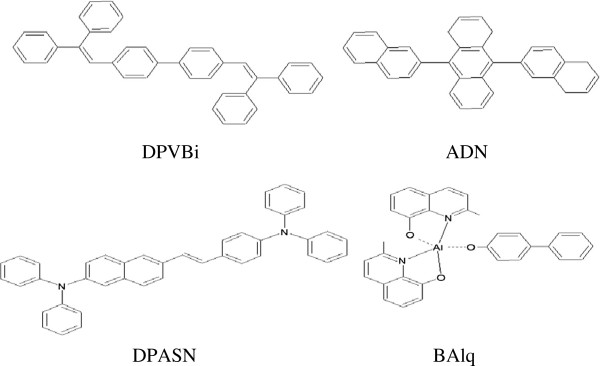
Molecular structures of different blue emissive materials used in this work.

Figure 1 shows the molecular structures of the different blue chromophores used in the OLED devices. We fabricated two types of blue OLEDs. The first type has a conventional device structure of ITO/NPB/DPVBi or ADN or DPASN/BAlq/Bphen/Liq/Al, where ITO, NPB, DPVBi (or ADN or DPASN), and Al are the anode, hole transporting layer, emissive layer, electron transporting layer, and cathode, respectively. The other type of blue OLEDs with a structure of ITO/NPB/DPVBi or ADN or DPASN/BAlq/DPVBi or ADN or DPASN/Bphen/Liq/Al was also fabricated for comparison studies. A list of OLEDs with different layer structures is summarized in Table 1.

**Table 1:** Layer structures of OLED devices A, B, C, and D

	**Structure**
Device A	ITO (1,800 Ǻ)/NPB (700 Ǻ)/DPVBi (300 Ǻ)/Bphen (300 Ǻ)/Liq (20 Ǻ)/Al (1,200 Ǻ)
Device B	ITO (1,800 Ǻ)/NPB (700 Ǻ)/ADN (300 Ǻ)/Bphen (300 Ǻ)/Liq (20 Ǻ)/Al (1,200 Å)
Device C	ITO (1,800 Ǻ)/NPB (700 Ǻ)/DPASN (300 Ǻ)/Bphen (300 Ǻ)/Liq (20 Ǻ)/Al (1,200 Ǻ)
Device D	ITO (1,800 Ǻ)/NPB (700 Ǻ)/BAlq (300 Ǻ)/Bphen (300 Ǻ)/Liq (20 Ǻ)/Al (1,200 Ǻ)
Device E	ITO (1,800 Ǻ)/NPB (700 Ǻ)/DPVBi (100 Ǻ)/BAlq (100 Ǻ)/DPVBi (100 Ǻ)/Bphen (300 Ǻ)/Liq (20 Ǻ)/Al (1,200 Ǻ)
Device F	ITO (1,800 Ǻ)/NPB (700 Ǻ)/ADN (100 Ǻ)/BAlq (100 Ǻ)/ADN (100 Ǻ)/Bphen (300 Ǻ)/Liq (20 Ǻ)/Al (1,200 Ǻ)
Device G	ITO (1,800 Ǻ)/NPB (700 Ǻ)/DPASN (100 Ǻ)/BAlq (100 Ǻ)/DPASN (100 Ǻ)/Bphen (300 Ǻ)/Liq (20 Ǻ)/Al (1,200 Ǻ)

With various DC voltage bias, the optical and electrical properties of blue OLEDs such as the current density, luminance, power efficiency, luminous efficiency, Commission Internationale deL'eclairage (CIExy) coordinates, and electroluminescence spectra were measured with Keithley 238 (Seoul, Korea), LMS PR-650 spectrophotometer and colorimeter (Photo Research Inc., CA, USA) and the IVL system (LMS Inc., Gyeonggi-do, Korea).

## Results and discussion

**Figure 2:**
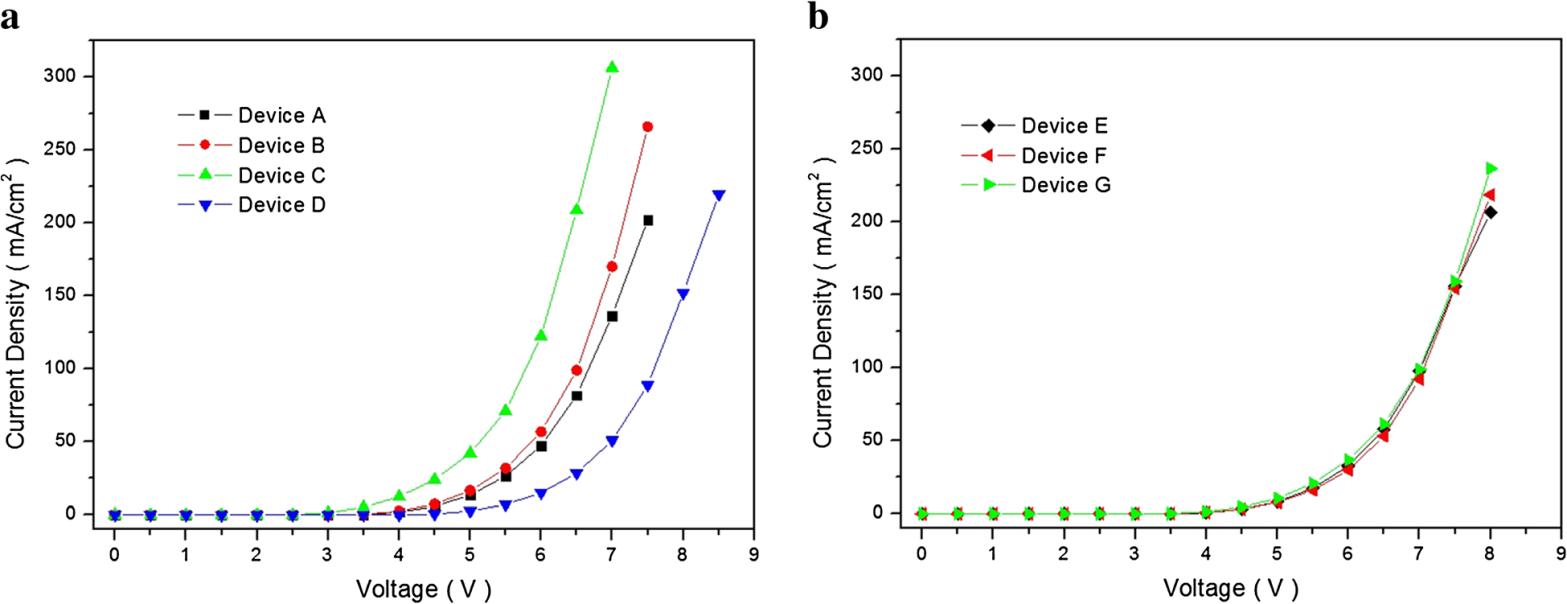
**Current density-voltage characteristics.** Measured for **(a)** conventional blue OLED devices A, B, C, and D and **(b)** OLEDs E, F, and G with QWS multiple emissive layers.

**Figure 3:**
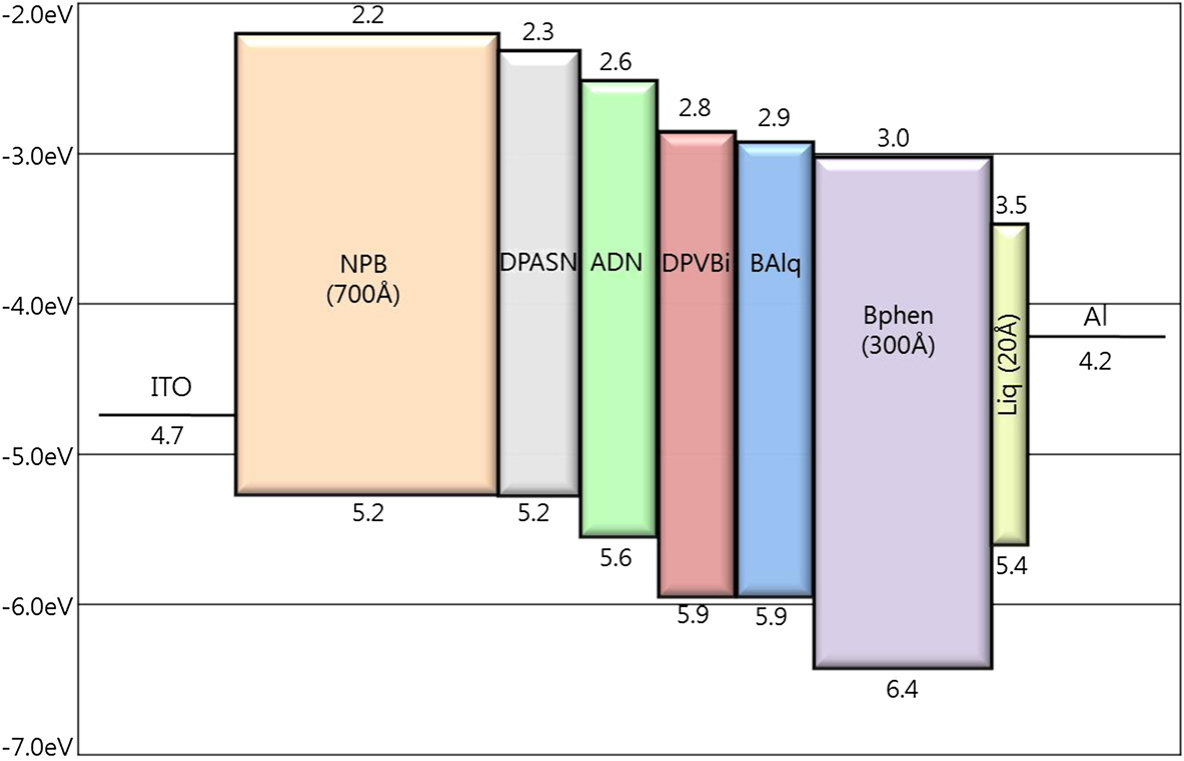
HOMO-LUMO energy levels of the functional organic materials used in the device fabrication.

Figure 2a shows the current density-voltage characteristics measured for each conventional blue OLED devices. Device C has the highest current density, and its EML consists of DPASN which is a p-type emitting material with a higher hole mobility; device B, which also had a p-type material, shows the second highest current density device. However, device D with an n-type emitting material of BAlq shows the lowest current density. Consequently, we realized that a p-type semiconductor has more electron affinity than a n-type semiconductor [24]. Figure 2b shows the current density-voltage characteristics measured for each QWS triple emissive layer blue OLED device. Devices E, F, and G actually have similar current density characteristic, and this phenomenon is caused by different charge injection barriers between emitting materials. The energy band diagrams of devices A to G are shown in Figure 3. Although it is not so significant to compare other QWS blue OLED devices, the device G including DPASN shows the highest current density at 8 V because the hole and electron injection barriers of device G were higher than those of another devices. As a result, the charge flow of charge injection barriers are interrupted, in turn decreasing its current density. Current densities of QWS blue OLED devices E, F, and G were lower than that of conventional OLED devices A, B, and C because electrons and holes are confined in the QWS which could possibly inhibit the current flow in EML.

**Figure 4:**
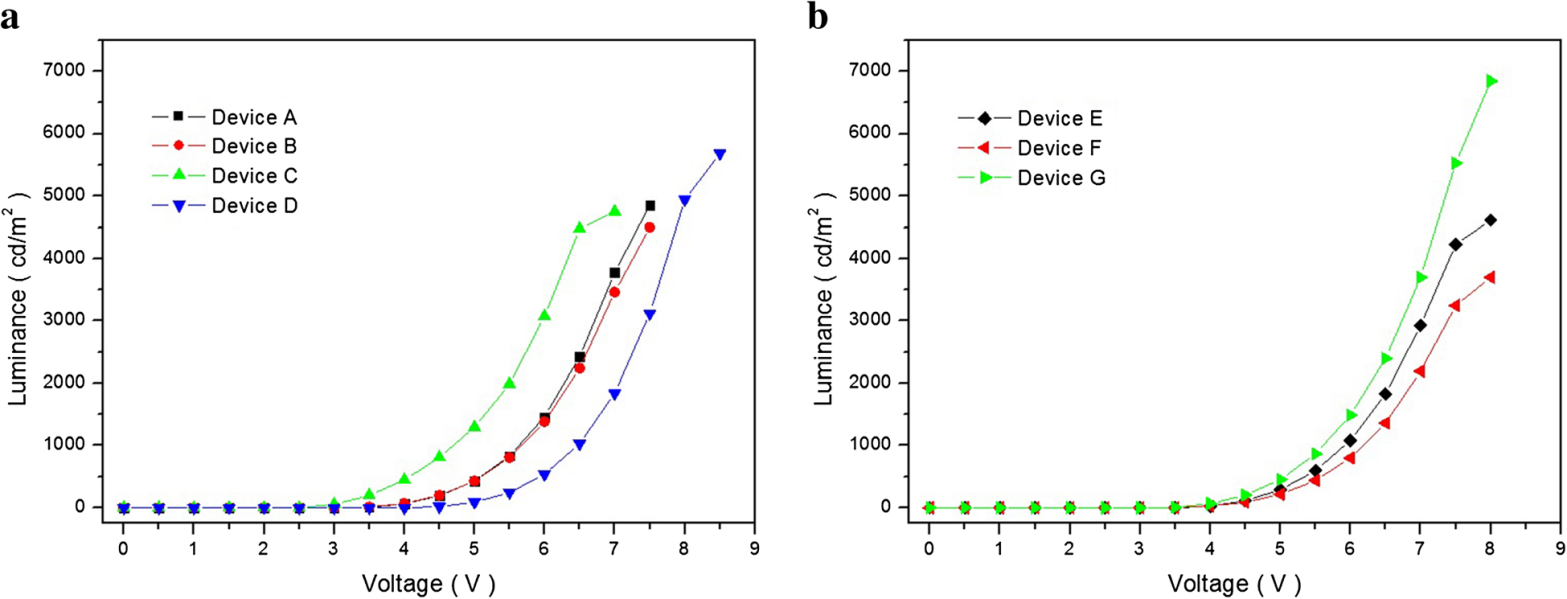
**Luminance-voltage characteristics. (a)** Conventional OLED devices A, B, C, and D and **(b)** QWS OLED devices E, F, and G.

Figure 4a,b shows the luminance-voltage (*L*-*V*) characteristics of conventional blue OLEDs and QWS multi-emissive layer blue OLEDs. Conventional blue OLEDs have higher luminance than QWS blue OLEDs. This is because QWS blue OLEDs consist of p-type emissive materials of DPVBi, ADN, and DPASN, and n-type emissive material of BAlq together. Consequently, n-type emissive materials influence on luminance much more than p-type emissive material although p-type emissive materials tend to have a higher luminance characteristic. Table 2 summarizes the luminance of each blue OLED device measured at 5, 6, and 7 V.

**Table 2:** Luminance of OLED devices measured at 5 to 7 V

	**5 V**	**6 V**	**7 V**
Device A	432.5 cd/m^2^	1,461 cd/m^2^	3,777 cd/m^2^
Device B	431.3 cd/m^2^	1,384 cd/m^2^	3,460 cd/m^2^
Device C	1,296 cd/m^2^	3,071 cd/m^2^	4,750 cd/m^2^
Device D	89.17 cd/m^2^	538.6 cd/m^2^	1,840 cd/m^2^
Device E	291.6 cd/m^2^	1,083 cd/m^2^	2,925 cd/m^2^
Device F	216.9 cd/m^2^	801.1 cd/m^2^	2,192 cd/m^2^
Device G	454.9 cd/m^2^	1,489 cd/m^2^	3,696 cd/m^2^

**Figure 5:**
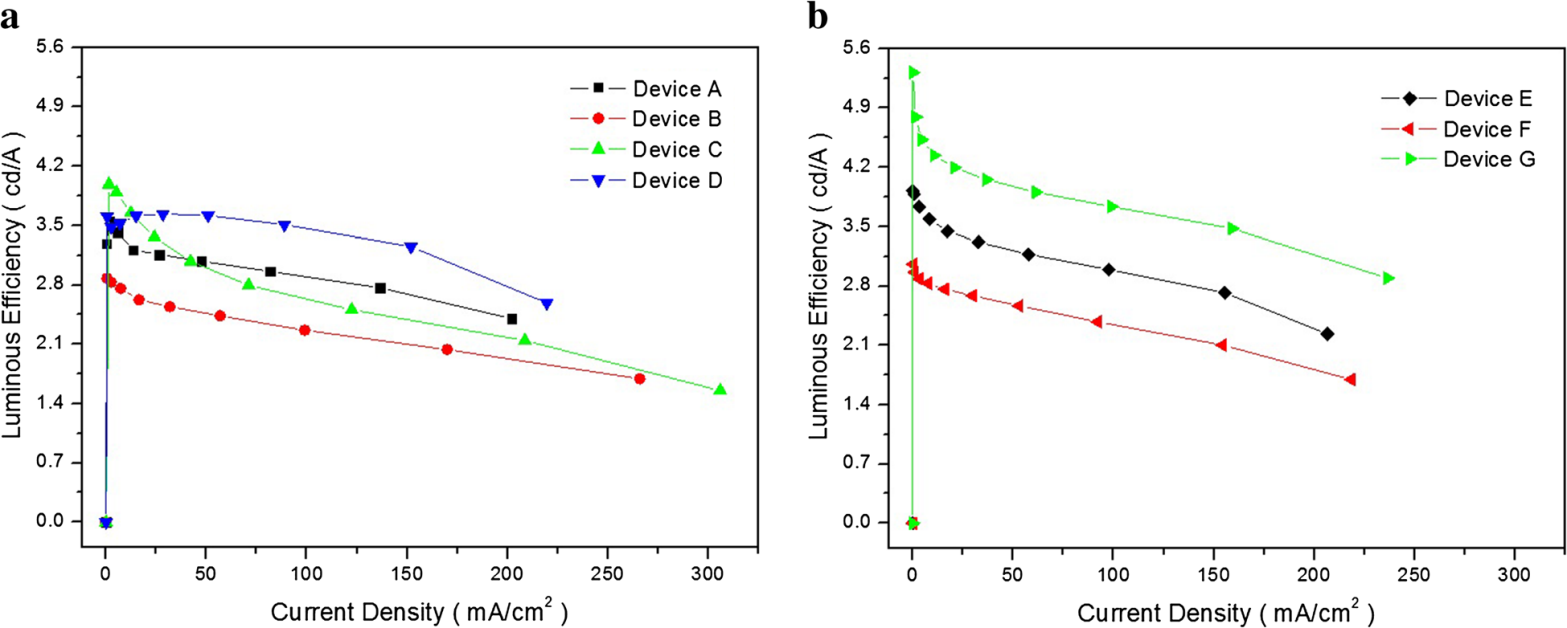
**Luminous efficiencies. (a)** Conventional OLED devices A, B, C, and D and **(b)** QWS OLED devices E, F, and G as a function of the current density.

Figure 5a,b shows the plot of luminous efficiency versus current density of conventional blue OLED device and QWS multi-emissive blue OLED devices. Table 3 summarizes the luminous efficiency of each device ranging from 50 to 150 mA/cm^2^. Luminous efficiency of QWS blue OLED devices is higher than that of conventional OLED devices. This phenomenon caused by emissive region of QWS OLED was evenly formed by DPVBi, ADN, DPASN, and BAlq. As a result, the power efficiency was enhanced because ADN and BAlq were emissive at different wavelengths. However, devices including DPASN show remarkable enhancement of efficiency. This can be explained by depth of QWS according to the difference of HOMO-LUMO energy level of emissive materials. HOMO and LUMO difference of DPVBi, BAlq, AND, and BAlq was 0, 0.1, 0.3, and 0.3 eV, respectively. This HOMO and LUMO level difference is not enough to confine charges and excitons in the emissive layer. Therefore, it was not enough to enhance luminous efficiency of OLED devices. However, when DPASN was used, luminous efficiency remarkably improved because HOMO and LUMO level difference of QWS OLED device was 0.7 and 0.6 eV between DPASN and BAlq. Therefore, QWS OLED devices need enough intermolecular HOMO and LUMO level difference of more than at least 0.3 eV.

**Table 3:** **Luminous efficiency of OLED devices measured at different current densities of 50 to 150 mA/cm**^**2**^

	**50 mA/cm**^**2**^	**100 mA/cm**^**2**^	**150 mA/cm**^**2**^
Device A	3.01 cd/A	2.86 cd/A	2.57 cd/A
Device B	2.48 cd/A	2.19 cd/A	2.14 cd/A
Device C	2.93 cd/A	2.65 cd/A	2.31 cd/A
Device D	3.62 cd/A	3.38 cd/A	3.31 cd/A
Device E	3.24 cd/A	2.85 cd/A	2.84 cd/A
Device F	2.62 cd/A	2.23 cd/A	2.21 cd/A
Device G	3.97 cd/A	3.64 cd/A	3.60 cd/A

**Figure 6:**
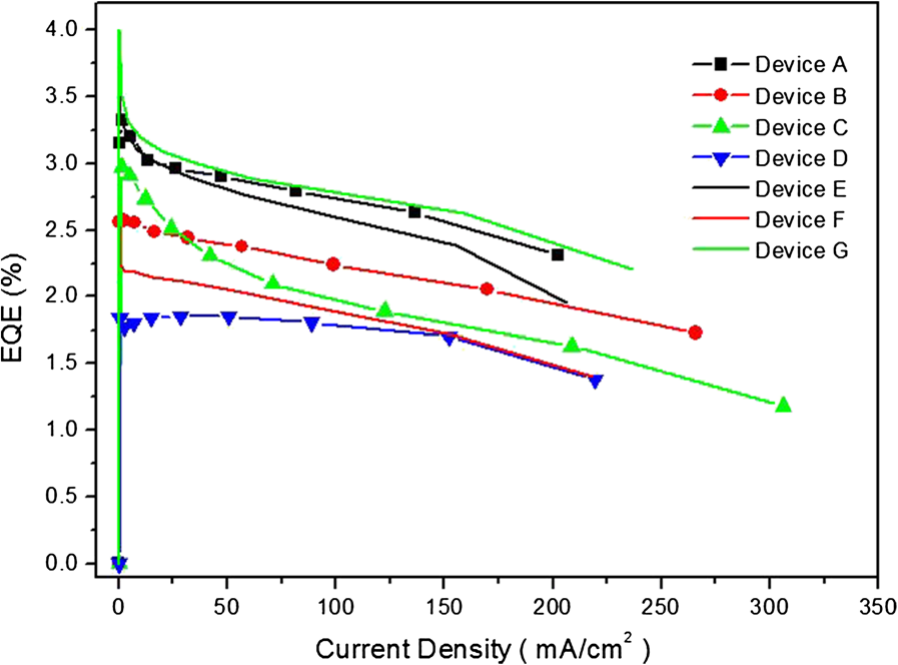
External quantum efficiency-current density characteristics measured for conventional OLEDs and QWS OLEDs.

The depth of QWS according to the difference of HOMO-LUMO energy level of emissive materials was concerned with the performance of the OLED. It is shown that the performance of OLEDs changes according to the depth of QWS (Figure 6). Figure 6 shows the plot of external quantum efficiency (EQE) as a function of current density for conventional OLEDs and QWS OLEDs. EQEs of OLED devices measured at 100 mA/cm^2^ were 2.71%, 2.21%, 1.99%, 1.75%, 2.53%, 1.81%, and 2.76%, respectively. QWS OLEDs having DPASN demonstrated a 38% enhancement in EQE. However, if QWS OLED devices include ADN and DPVBi, the EQE did not change or decrease. As mentioned before, when using DPASN, EQE enhances because the depth of QWS OLED device is enough for 0.7 and 0.6 eV. If QWS OLED devices include ADN and DPVBi, the depth of QWS was not enough to enhance EQE, and the emission region was formed at BAlq with lower EQE. Therefore, the EQE of OLED devices was decreased.

**Figure 7:**
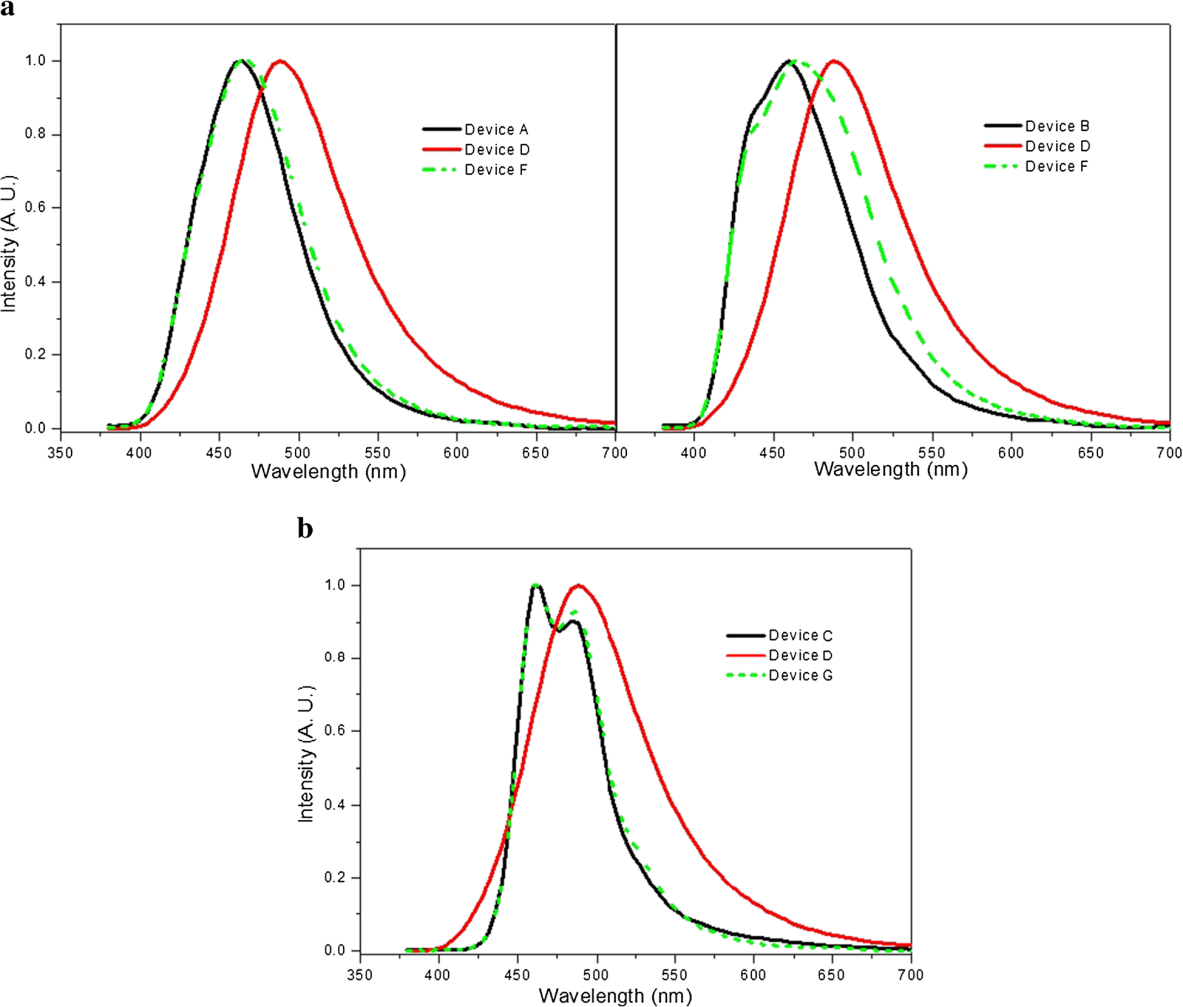
A comparison of EL spectra measured for conventional OLEDs and QWS OLEDs at 5 V (a, b).

Figure 7 shows the EL spectra of conventional OLED devices and QWS OLED devices at 5 V. We know that if QWS OLED devices include ADN and DPVBi, the full width at half maximum (FWHM) of EL spectra was increased. We can observe this result in Figure 7a,b. As the result demonstrates, the emission region formed at BAlq. However, when using DPASN at QWS OLED, the EL spectra remained almost the same.

## Conclusions

Blue OLED with triple emissive layer structure achieved luminous efficiency of 5.23 cd/A at 3.5 V, which is 36% higher than that of the conventional blue OLEDs. Obviously, the quantum well-like structure is favorable for hole-electron recombination for efficient exciton generation in the multiple emissive layers of DPVBi, ADN, and DPASN with BAlq in the device. There was no significant improvement in the luminous efficiency (only about 3% and 4%) when DPVBi and ADN were used as the additional emitting layer to form a quantum well-like structure; a 36% improvement in luminous efficiency was realized in DPASN/BAlg/DPASN blue OLEDs. This result shows that blue OLEDs can only improve luminous efficiency under proper difference in HOMO and LUMO energy level between the central and surrounding emitting layers. The effect of layer thickness and combination of different emissive layers on charge carrier transport mechanism from the quantum well-like and the blue emitting layer based on space charge limited current will be further examined.

## Competing interests

The authors declare that they have no competing interests.

## Authors’ contributions

JY and YK conceived and designed the experiments. JY and NHK carried out the experiments with contributions from SYL. FRZ designed and synthesized the materials of OLEDs. SIY carried out the characterization of devices. YK supervised the work. JY and WYK wrote the manuscript. All authors read and approved the final manuscript.

## References

[B1] Tang CW VanSlyke SA Organic electroluminescent diodes Appl Phys Lett 1987 9 913

[B2] Tatsuo M Takaaki I Teruyoshi Mizutani J Electroluminescent properties of organic light-emitting diodes with blue-emitting Alq Photopolym Sci Technol 2004 9 2

[B3] Reineke S Lindner F Schwartz G Seidler N Walzer K Lüssem B Leo K White organic light-emitting diodes with fluorescent tube efficiency Nature 2009 9 234 19444212 10.1038/nature08003

[B4] Gu G Burrows PE Venkatesh S Forrest SR Thompson ME Vaccum-deposited, nonpolymeric flexible organic light-emitting devices Opt Lett 1997 9 172 18183139 10.1364/ol.22.000172

[B5] Dandan S Suling Z Hany A Modification of exciton lifetime by the metal cathode in phosphorescent OLEDs, and implications on device efficiency and efficiency roll-off behavior Adv Funct Mater 2011 9 2311

[B6] Choi WH Tam HL Zhu FR Ma DG Sasabe H Kido J High performance semitransparent phosphorescent white organic light emitting diodes with bi-directional and symmetrical illumination Appl Phys Lett 2013 9 153308

[B7] Chen YH Chen JS Ma DG Yan DH Wang LX Zhu FR High power efficiency tandem organic light-emitting diodes based on bulk heterojunction organic bipolar charge generation layer Appl Phys Lett 2011 9 243309

[B8] D'Andrade BW Forrest SR White organic light-emitting devices for solid-state lighting Adv Mater (Weinheim, Ger) 2004 9 1585

[B9] Krummacher BC Choong VE Mathai MK Choulis SA So F Jermann F Fiedler T Zachau M Highly efficient white organic light-emitting diode Appl Phys Lett 2006 9 113506

[B10] D'Andrade BW Holmes RJ Forrest SR Efficient organic electrophosphorescent white-light-emitting device with a triple doped emissive layer Adv Mater (Weinheim, Ger) 2004 9 624

[B11] Shinar J Organic Light-Emitting Devices 2004 New York: Springer

[B12] Gautier-Thianche E Sentein C Lorin A Denis C Raimond P Nunzi JM Effect of coumarin on blue light-emitting diodes based on carbazol polymers J Appl Phys 1998 9 4236

[B13] Hubert C Fiorini-Debuisschert C Hassiaoui I Rocha L Raimond P Nunzi JM Emission properties of an organic light-emitting diode patterned by a photoinduced autostructuration process Appl Phys Lett 2005 9 191105

[B14] Zang FX Sum TC Huan ACH Li TL Li WL Zhu FR Reduced efficiency roll-off in phosphorescent organic light emitting diodes at ultrahigh current densities by suppression of triplet-polaron quenching Appl Phys Lett 2008 9 023309

[B15] Kim SH Jang J Hong JM Lee JY High efficiency phosphorescent organic light emitting diodes using triplet quantum well structure Appl Phys Lett 2007 9 173501

[B16] Liu SM Li B Zhang LM Song H Jiang H Enhanced efficiency and reduced roll-off in nondoped phosphorescent organic light-emitting devices with triplet multiple quantum well structures Appl Phys Lett 2010 9 083304

[B17] Zhao J Junsheng Y Zhang L Wang J Non-doped phosphorescent white organic light-emitting devices with a quadruple-quantum-well structure Physica B 2012 9 2753

[B18] Ohmori Y Fujii A Uchida M Morishima C Yoshino K Fabrication and characteristics of 8‒hydroxyquinoline aluminum/aromatic diamine organic multiple quantum well and its use for electroluminescent diode Appl Phys Lett 1993 9 3250

[B19] Qiu Y Gao Y Wang L Wei P Duan L Zhang D Dong G High-efficiency organic light-emitting diodes with tunable light emission by using aromatic diamine/5,6,11,12-tetraphenylnaphthacene multiple quantum wells Appl Phys Lett 2002 9 3540

[B20] Qiu Y Gao Y Wei P Wang L Organic light-emitting diodes with improved hole-electron balance by using copper phthalocyanine/aromatic diamine multiple quantum wells Appl Phys Lett 2002 9 2628

[B21] Song SF Zhao DW Xu Z Xu XR Energy transfer in organic quantum well structures Acta Phys Sin 2007 9 3499

[B22] Zhu HN Xu Z Zhao SL Zhang FJ Kong C Yan G Gong W Influence of well structure on efficiency of organic light-emitting diodes Acta Phys Sin 2010 9 8093

[B23] Jian Z Juan G Zhuo G Ke D Jiule C An organic light-emitting device with ultrathin quantum-well structure as light emitting layer Opt Rev 2011 9 394

[B24] Culligan W Chen AC-A Wallace JU Klubek KP Tang CW Chen SH Effect of hole mobility through emissive layer on temporal stability of blue organic light-emitting diodes Adv Funct Mater 2006 9 1481

